# Ecological Momentary Assessment of Midlife Adults’ Daily Stress: Protocol for the Stress Reports in Variable Environments (STRIVE) App Study

**DOI:** 10.2196/51845

**Published:** 2023-10-05

**Authors:** Evan J Jordan, Patrick C Shih, Erik J Nelson, Stephen J Carter, Mario Schootman, Aric A Prather, Xing Yao, Chasie D Peters, Canaan S E Perry

**Affiliations:** 1 Department of Health and Wellness Design School of Public Health - Bloomington Indiana University Bloomington, IN United States; 2 Luddy School of Informatics, Computing, and Engineering Indiana University Bloomington, IN United States; 3 Department of Public Health Brigham Young University Provo, UT United States; 4 Department of Kinesiology School of Public Health - Bloomington Indiana University Bloomington, IN United States; 5 College of Medicine University of Arkansas for Medical Sciences Little Rock, AR United States; 6 Department of Psychiatry and Behavioral Sciences Weill Institute for Neurosciences University of California San Francisco, CA United States; 7 Regenstrief Institute Indianapolis, IN United States

**Keywords:** activity trackers, built environment, ecological momentary assessment, heart rate monitoring, life stress, physical activity, spatial analysis, wearable technology

## Abstract

**Background:**

Daily stressors are associated with cognitive decline and increased risk of heart disease, depression, and other debilitating chronic illnesses in midlife adults. Daily stressors tend to occur at home or at work and are more frequent in urban versus rural settings. Conversely, spending time in natural environments such as parks or forests, or even viewing nature-themed images in a lab setting, is associated with lower levels of perceived stress and is hypothesized to be a strong stress “buffer,” reducing perceived stress even after leaving the natural setting. However, many studies of daily stress have not captured environmental contexts and relied on end-of-day recall instead of in-the-moment data capture. With new technology, these limitations can be addressed to enhance knowledge of the daily stress experience.

**Objective:**

We propose to use our novel custom-built Stress Reports in Variable Environments (STRIVE) ecological momentary assessment mobile phone app to measure the experience of daily stress of midlife adults in free-living conditions. Using our app to capture data in real time will allow us to determine (1) where and when daily stress occurs for midlife adults, (2) whether midlife adults’ daily stressors are linked to certain elements of the built and natural environment, and (3) how ecological momentary assessment measurement of daily stress is similar to and different from a modified version of the popular Daily Inventory of Stressful Events measurement tool that captures end-of-day stress reports (used in the Midlife in the United States [MIDUS] survey).

**Methods:**

We will enroll a total of 150 midlife adults living in greater Indianapolis, Indiana, in this study on a rolling basis for 3-week periods. As those in underrepresented minority groups and low-income areas have previously been found to experience greater levels of stress, we will use stratified sampling to ensure that half of our study sample is composed of underrepresented minorities (eg, Black, American Indian, Hispanic, or Native Pacific Islanders) and approximately one-third of our sample falls within low-, middle-, and high-income brackets.

**Results:**

This project is funded by the National Institute on Aging from December 2022 to November 2024. Participant enrollment began in August 2023 and is expected to finish in July 2024. Data will be spatiotemporally analyzed to determine where and when stress occurs for midlife adults. Pictures of stressful environments will be qualitatively analyzed to determine the common elements of stressful environments. Data collected by the STRIVE app will be compared with retrospective Daily Inventory of Stressful Events data.

**Conclusions:**

Completing this study will expand our understanding of midlife adults’ experience of stress in free-living conditions and pave the way for data-driven individual and community-based intervention designs to promote health and well-being in midlife adults.

**International Registered Report Identifier (IRRID):**

DERR1-10.2196/51845

## Introduction

Daily stressors are associated with cognitive decline and increased risk of heart disease, depression, and other debilitating chronic illnesses for midlife adults [1-5]. Daily stressors tend to occur at home or at work and are more frequent in urban versus rural settings [6-8]. Conversely, spending time in natural environments such as parks or forests, or even viewing nature-themed images in a lab setting, is associated with lower levels of perceived stress and is hypothesized to be a strong stress “buffer,” reducing perceived stress even after leaving the natural setting [9-12]. The role that natural environments can play in reducing stress arousal is particularly important when considering that neighborhoods with high poverty rates and higher proportions of racial or ethnic minorities have less access to green space and higher exposure to stressful elements such as abandoned buildings and crime [13-15]. However, many studies of daily stress have not captured environmental contexts and relied on end-of-day recall instead of in-the-moment data capture. With new technology, these limitations can be addressed to enhance knowledge of the daily stress experience [16].

In the United States, midlife adults are staying in the workforce longer and are engaging in higher levels of physical activity than previous generations, potentially extending their interactions with elements of the built and natural environments that influence their experience of daily stress [17,18]. Recent evidence has revealed that age-related linear increases in perceptions of well-being observed during past periods of economic prosperity have not continued during more recent, less economically prosperous times [19]. The seismic and unpredictable economic and social changes brought about by the COVID-19 pandemic have greatly altered the experience of daily stressors in the built and natural environment, a trend that is likely to continue well into the future [20].

In the midlife adult population, stress (or the lack thereof) plays an important role in the aging process. The relationship between various types of stress and alterations in cognitive functioning has been well documented, and there is growing evidence showing a relationship between daily stress in midlife and cognitive decline [2,3,21]. Perceived stress in midlife adults has also been closely linked with physical disability later in life [22,23]. A growing body of research has also found that perceived stress, particularly in the workplace, is closely related to heart disease [24]. Furthermore, while individual health and wellness in midlife is no doubt important, longitudinal studies have shown that the health and wellness of midlife adults is also pivotal to others in their workplace, society in general, and in the lives of those in younger and older generations who often are socioeconomically dependent on midlife family members [25].

Midlife adults of different socioeconomic statuses and races are subject to structural inequality in their living environments. Those living in urban environments with higher levels of poverty and violence experience higher levels of environmental stress than those living in suburban or rural environments [15]. In particular, it is well documented that underrepresented minorities are exposed to environmental stressors more than nonunderrepresented minorities [26,27]. One explanation for racial differences in exposure to stressors is residential segregation, resulting in a predisposition for low-income minorities’ exposure to environmental stressors such as traumatic events, urban decay, and poverty [28]. Minority and low-income midlife adults’ exposure to environmental stressors has been linked to unhealthy behaviors like cigarette smoking [29], as well as myriad negative health outcomes [30].

In response to this research gap, we propose to use our novel custom-built Stress Reports in Variable Environments (STRIVE) ecological momentary assessment (EMA) mobile phone app to measure the experience of daily stress of midlife adults in free-living conditions. Using our app to capture data in real time will allow us to determine (1) where and when daily stress occurs for midlife adults, (2) whether midlife adults’ daily stressors are linked to certain elements of the built and natural environment, and (3) how EMA measurement of daily stress is similar to and different from a modified version of the popular Daily Inventory of Stressful Events (DISE) measurement tool that captures end-of-day stress reports (used in the Midlife in the United States [MIDUS] survey) [31].

Completing this study will expand our understanding of mid-life adults’ experience of stress in free-living conditions and pave the way for data-driven individual and community-based intervention designs to promote health and well-being in midlife adults. Potential interventions that could stem from our data include technology-based “just-in-time” interventions that account for spatiotemporal location, mindfulness-based interventions that incorporate environmental stress triggers, and community-based interventions designed to eliminate environmental stressors that are commonly experienced by midlife adults (eg, congested areas).

## Methods

### Overview

In this stage 0 (of the NIH Stage Model for behavioral intervention development) study, we propose an interdisciplinary mixed method approach to examine the occurrence of daily stressors in the moment they occur for midlife adults in the built and natural environment using mobile and wearable technology. A total of 150 midlife adults will be enrolled in this study on a rolling basis for 3-week periods. Participants will meet with a researcher in person at the start and end of their 3-week study period. They will be provided with a smartphone preloaded with the STRIVE app and a Polar Unite smartwatch. They will be instructed to carry the phone and wear the watch for the 3-week duration of the study. Each enrolled participant will use one or more daily stress measurement tools in any given week, randomized to account for participant priming. In 1 week of participation, participants will complete the DISE by the Research Electronic Data Capture (REDCap; Vanderbilt University) survey every evening. In another week of participation, participants will use the STRIVE app to report daily stressors in real time and complete the DISE every evening. In another week of participation, participants will only report stressors in real time through the STRIVE app. Automated SMS text messages will be sent to participants each day to remind them of what they need to do to remain active in the study and provide survey links when needed.

Participants will be recruited from the greater Indianapolis area in Indiana. As those in underrepresented minority groups and low-income areas have previously been found to experience greater levels of stress, we will use stratified sampling to obtain a sample that is racially and socioeconomically diverse (Table 1). Income levels are defined as the bottom, middle, and top third of household incomes based on 2020 US Census percentiles (low income: <US $39,535; middle income: US $39,535-$85,076; high income: >US $85,076) [32]. Participants will be recruited with the assistance of the Indiana Clinical and Translational Sciences Institute recruitment concierge services and their community partnerships. Recruitment materials will be posted on community-based social media pages, on paper flyers in public community spaces, and through all communication channels operated by community health partners.

**Table 1:** Study sample stratified by race and household income (N=150).

Race	Low income, n (%)	Middle income, n (%)	High income, n (%)
Black, American Indian, Hispanic, Native Pacific Islanders	25 (16.6)	25 (16.6)	25 (16.6)
White or Asian	25 (16.6)	25 (16.6)	25 (16.6)

### Measurement of Stress With the STRIVE App

**Figure 1:**
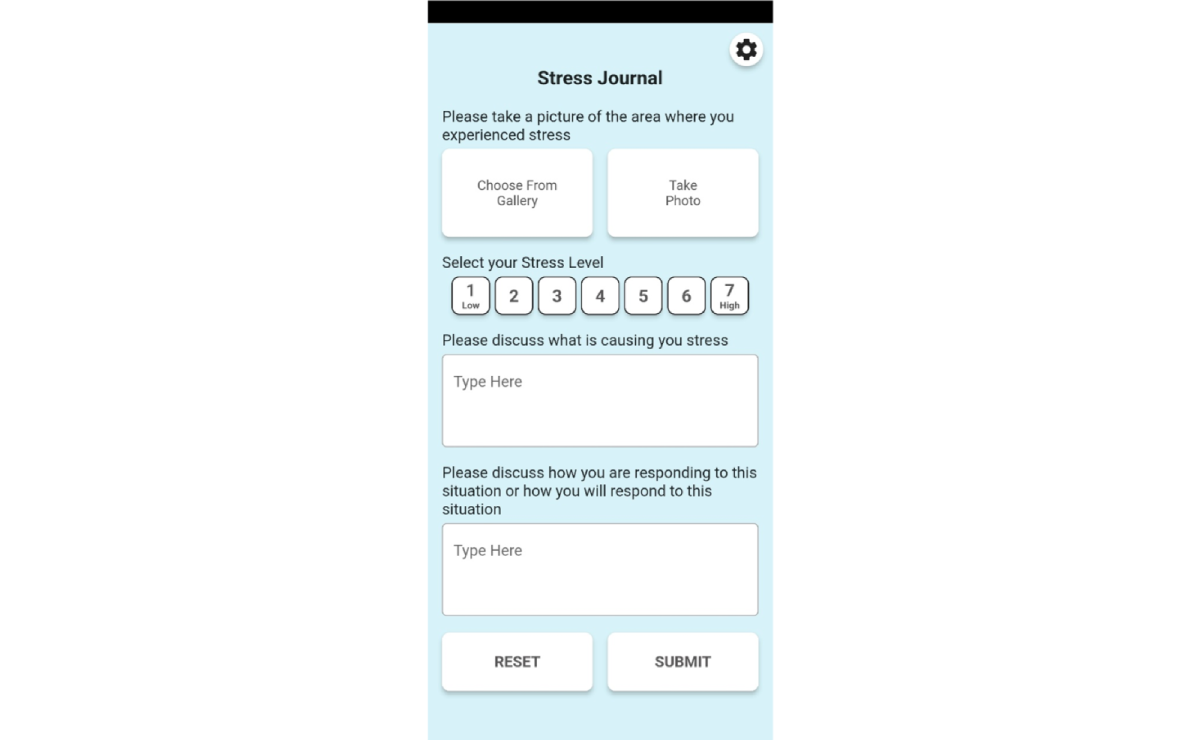
The Stress Reports in Variable Environments (STRIVE) app.

Study participants will be asked to carry the researcher’s provided Android mobile phone with them at all times. The STRIVE app passively records the participant’s GPS coordinates every 60 seconds throughout the data collection period (Table 2). Research participants are informed that their location is being tracked by the app. When participants experience a daily stressor, they are asked to open the app and provide a rating of their stress level, a description of the stressor, their preferred coping strategy, and optionally take a photograph of the environment where they experienced stress (Figure 1). Data input is kept to a minimum to reduce participant burden and burnout [33].

**Table 2:** All study measures.

Measurement tools			Measure
**Pretest survey**			
	CDC^a^ Healthy Days [34]	Overall health	
	Godin Leisure-Time Exercise Questionnaire [35]	Physical activity level	
	Portion of Behavioral Risk Factor Surveillance (BRFSS) [36]	Substance use and abuse	
	Social Provisions Scale [37]	Social support	
	UCLA^b^ Loneliness Scale [38]	Loneliness	
	Patient Health Questionnaire-9 (PHQ-9) [39]	Depressive symptoms	
	Adverse Childhood Events Scale (ACES) [40]	Early life stress	
	Economic Strain Model [41]	Financial strain	
	Neighborhood Disorder Scale [42]	Neighborhood safety	
**Real time measurements (all time-stamped)^c^**			
	STRIVE^d^ app	Per 60 second GPS coordinates (continuous) Self-reported stress level (7-point scale) Self-reported stressor (text-based) Self-reported coping response (text-based) Photo of stressful environment (JPEG)	
	Polar Unite Smartwatch	Heart rate (continuous) Activity level (continuous)	
End-of-day recall DISE^e^ measurements^c^			Self-reported overall stress level (7-point scale) Self-reported stressor type Self-reported stress level by stressor (7-point scale) Self-reported date

^a^CDC: Centers for Disease Control and Prevention.

^b^UCLA: University of California, Los Angeles.

^c^Additional measurements include demographics and health history.

^d^STRIVE: Stress Reports in Variable Environments.

^e^DISE: Daily Inventory of Stressful Events.

We have created a protocol to promote the safety of research participants during their reporting of daily stressors with the STRIVE app. If they cognitively appraise themselves as stressed and are in a situation in which it is unsafe to use their phones (eg, driving a car), they are asked to proceed to the nearest safe location before recording their experience. Participants will be asked to indicate when and where the stressor occurred if they are not reporting from the location in which the stress occurred in their later description of it. Many people go through a day without experiencing a stressor [43]; for days when this is the case, participants will be asked to indicate on their app that they had not experienced stress but were still actively participating in the study.

### Measurement of Stress With the Modified DISE Tool

The DISE is recommended by the National Institute on Aging funded Stress Measurement Network for daily stress measurement. A modified version of the DISE will be administered through the REDCap survey each evening for 2 weeks of the study period. The modified version of the DISE includes questions about the number of times participants felt stressed over the course of the day, their overall stress level, and questions about specific stressor categories (eg, had an argument and had work stress) and how stressful each of those experiences was for the participant. The DISE was modified to allow for direct comparisons between STRIVE and DISE.

### Geospatial Analysis

We will fit spatiotemporal regression models to determine the impact of specific elements of the built and natural environment on daily stressors. Given the nature of our spatially dependent data, traditional regression-based approaches (eg, Poisson or negative binomial regression) violate the independence assumption required for generalized linear models. Ignoring the dependence of spatial data can underestimate standard errors, resulting in overly narrow confidence interval estimates and, consequently, incorrect statistical inference [44]. Thus, we will use spatiotemporal models that can simultaneously account for dependence (autocorrelation) in geospatial locations and temporal measurements. We will do this using Integrated Nested Laplace Approximation (INLA) models. Specifically, we will fit zero-inflated negative binomial INLA models, as these models can account for the many instances where stress experiences are not recorded (zero inflation) while still adjusting for the spatial and temporal autocorrelation of each measurement [45,46]. Importantly, the INLA modeling approach is robust as it can incorporate individual-level characteristics and group-level (ie, neighborhood) characteristics while adjusting for the spatial and temporal autocorrelation [47]. INLA uses a Bayesian paradigm, can fit offsets for any necessary weightings (eg, population size or number of measurements), is able to handle both point process (ie, singular latitude and longitude locations) and irregular lattice data (ie, polygons of parks and census tracts), and can analyze the individual impact of temporal, spatial, and spatiotemporal effects [46]. Through this analysis, we can identify the magnitude, direction, and impact of participant attributes (including data collected through the pretest survey and real-time collection of heart rate and activity level data from the Polar Unite watch), neighborhood features, and location characteristics on daily stressors.

### Qualitative Analysis of Reported Stressors

We will conduct an analysis of data reported through the STRIVE app in a 2-step process including open and axial coding [48]. Open coding will be conducted twice: first to code stressors into stressor types and second to code mentions of elements of the built and natural environment that played a role in the stressful experience. This open coding process will be conducted on qualitative responses to the question, “Please discuss what is causing you stress.” In this open coding process, researchers will be versed in previous research categorizing daily stressors to identify themes in the data that may overlap with commonly experienced daily stressors identified in previous research [49]. Any new themes that emerge will be given new names and detailed descriptions. In the second axial coding step, researchers will analyze stressor and environmental characteristic themes to look for those that may be related to identify linkages, creating meta-categories for daily stressors that may be similar in nature. Then, the open and axial coding processes will be conducted on participant photographs that accompany stressor reports. Characteristics of the environment in which stress occurs will be coded for each participant photograph, allowing for triangulation among themes generated by text-based responses and participant photographs**.** This data triangulation will allow us to understand how daily stressors are related to specific elements of the built and natural environment.

### Comparison of STRIVE and DISE Stressor Data

We will compare the number of stressors reported, the type of stressors reported, and the subjective severity of stressors reported by the same individual using the DISE and STRIVE app across 3 time periods. The 3 time periods are designed to differentiate between stand-alone use of each instrument and concurrent use of both instruments, as there is potential for these instruments to be complementary in terms of the scope and depth of data captured. Thematic coding of stressors reported through the DISE and STRIVE app will be conducted to allow for comparison between the two. All stressors will be coded into 7 categories specified by the DISE to retain consistency. Stressful events are categorized into 1 of 7 broad classifications organized by interpersonal tensions, work or education, home, finances, health or accident, network, and miscellaneous. As the unit of analysis of the DISE is the “day,” we will conduct a repeated measures ANOVA to compare the number of stressors reported per day, the average subjective severity of stressors per day, and the type of stressors reported per day across the 3 modes of daily stress reporting, including an interaction of group (STRIVE or DISE) and mode (using only one or both together) to test if there is any effect on the reported stressors by use. A factor will also be included for the order receiving the conditions to account for carryover effects. Models will also be adjusted by demographic variables.

### Ethics Approval

This study has been approved by the Indiana University Institutional Review Board (study number 1910712088). Informed consent has been and will be obtained from all research participants. A special document outlining when and where it is appropriate and safe to take photographs of stressful environments will be provided to all research participants, with a focus on protecting the privacy of others. Further, a document highlighting that study participants are not to report stressors in dangerous situations (eg, while driving a car) will be provided. Although we will make efforts to deidentify study data as much as possible, certain elements of data collected (eg, pictures and GPS coordinates) could be used to identify participants. Participants are informed of this risk and may choose not to report stressors or take pictures in situations where they are concerned about their confidentiality. Each study participant will be paid US $150 for their participation in the study.

## Results

This study will take approximately 24 months to complete and will take place between December 2022 and November 2024. Enrollment began in August 2023 and is expected to be completed by July 2024. We expect the following study findings:

1. A better understanding of where and when stressors occur for midlife adults in free-living conditions. We hypothesize that the quantity of stressors reported, type of stressors reported, and subjective severity of stressors reported by midlife adults will differ by location (eg, at home vs at a park) and time (eg, morning vs evening) throughout the built and natural environment.
2. An understanding of specific elements of the built and natural environment that contribute to midlife adults’ daily stress. We hypothesize that distinct environmental characteristics (eg, abandoned buildings) will emerge as contributors to stress through the qualitative analysis of stressor descriptions and pictures of stressful environments.
3. An understanding of how the STRIVE-based EMA of daily stress is supplementary or complementary to the DISE. We hypothesize that, when reporting through the STRIVE app compared with when reporting through the DISE, midlife adults will report a greater number of daily stressors, a lower average stressor severity, and different types of stressors.

Throughout the study, we will present our research findings at relevant academic or professional conferences and publish peer-reviewed papers. We will also share our study findings with partner governmental organizations and nongovernmental organizations in the state of Indiana. These organizations will likely find our data useful for future community planning.

## Discussion

Daily stress contributes to myriad negative health outcomes. Midlife adults are staying in the workforce longer than previous generations and are likely subject to greater environmental stressors than in the past. Technological advances, including smartphones and wearable technology, allow us to measure stress in situ, painting a clearer picture of how stress is experienced in real time. Our custom-built STRIVE EMA app allows us to reduce recall bias and geospatially locate stressors while simultaneously collecting health data (eg, heart rate, sleep, and physical activity), allowing for a clear picture of the role the built and natural environment play in the daily stress of midlife adults.

Completing this study will expand our understanding of midlife adults’ experience of stress in free-living conditions and pave the way for data-driven individual and community-based intervention designs to promote health and well-being in midlife adults: (1) technology-based “just-in-time” therapeutic interventions that account for spatiotemporal location, (2) mindfulness-based interventions that incorporate environmental stress triggers and stimuli, and (3) community-based interventions designed to eliminate environmental stressors that are commonly experienced by many midlife adults (eg, abandoned buildings, areas that often become crowded or congested, or other yet undiscovered stressful elements of the environment).

## Appendix

Multimedia Appendix 1 Peer-review report from the Community Influences on Health Behavior Study Section, Healthcare Delivery and Methodologies Integrated Review Group, CENTER FOR SCIENTIFIC REVIEW (National Institutes of Health, USA).

## Abbreviations

DISE: Daily Inventory of Stressful Events
EMA: ecological momentary assessment
INLA: Integrated Nested Laplace Approximation
MIDUS: Midlife in the United States
REDCap: Research Electronic Data Capture
STRIVE: Stress Reports in Variable Environments

## Data Availability

The data sets generated or analyzed during this study will be available from the corresponding author on reasonable request.
