# Supplementary material for: Ecological Momentary Assessment of Midlife Adults’ Daily Stress: Protocol for the Stress Reports in Variable Environments (STRIVE) App Study
Source: JMIR Res Protoc. 2023 Oct 5;12:e51845. doi: 10.2196/51845 (PMC10587811; doi:10.2196/51845)
Supplement: Multimedia Appendix 1 [file resprot_v12i1e51845_app1.pdf]

SUMMARY STATEMENT
(Privileged Communication)

PROGRAM CONTACT:
Dr. Janine Simmons
301-443-6652
simmonsj@mail.nih.gov

Release Date: **06/28/2022**
Revised Date:

Application Number: **1 R21 AG077333-01A1**
Formerly: **1R21AG077333-01**

Principal Investigator

JORDAN, EVAN

Applicant Organization: TRUSTEES OF INDIANA UNIVERSITY

Review Group: **CIHB**
Community Influences on Health Behavior Study Section

Meeting Date: **06/06/2022**
Council: **OCT 2022**
Requested Start: **12/01/2022**
RFA/PA: **PA20-195**
PCC: **2BPDIJS**

Project Title: **Examining the daily stress of mid-life adults in the built and natural environment**

SRG Action: **Impact Score:22 Percentile:6 +**
Next Steps: **Visit https://grants.nih.gov/grants/next_steps.htm**
Human Subjects: 48-At time of award, restrictions will apply
Animal Subjects: 10-No live vertebrate animals involved for competing appl.
Gender: 1A-Both genders, scientifically acceptable
Minority: 1A-Minorities and non-minorities, scientifically acceptable
Age: 7A-Only Adults, scientifically acceptable

Project Year	Direct Costs Requested	Estimated Total Cost
1	175,000	295,394
2	100,000	168,796
TOTAL	275,000	464,190

ADMINISTRATIVE BUDGET NOTE: The budget shown is the requested budget and has not been adjusted to reflect any recommendations made by reviewers. If an award is planned, the costs will be calculated by Institute grants management staff based on the recommendations outlined below in the COMMITTEE BUDGET RECOMMENDATIONS section.

1R21AG077333-01A1 Jordan, Evan

COMMITTEE BUDGET RECOMMENDATIONS
PROTECTION OF HUMAN SUBJECTS UNACCEPTABLE

RESUME AND SUMMARY OF DISCUSSION: Using mobile and wearable technology to capture psychological and physiological experiences of stress, this revised application attempts to better understand when and where daily stress occurs among mid-life adults. The significance of the study lies in its potential to provide a more comprehensive understanding of where and when stress occurs for mid-life adults and inform interventions that seek to aid healthy aging by minimizing environmental stressors and promoting activities in healthy environments. The results would be useful, for example, for planners, community organizations, and health care providers. The panel was enthusiastic about the many strengths of the application, including very the comprehensive and innovative methods and tools; use of mobile and wearable technology to capture in-the-moment experiences, which eliminate recall bias of previously used methods; the solid analysis plan; the excellent recruitment plan; the well-qualified research team, which has extensive experience in studying adverse outcomes, especially neighborhood-based ones; and strong preliminary data. The application has been mostly responsive to the previous review comments, but few issues remain (e.g., generalizability). Also, letters of support from community-based organizations are missing. Some concerns were also noted around human subjects and the IRB. Overall, this is an impressive application from an exceptional group of investigators and enthusiasm was very high. The proposed research is viewed as being outstanding and could have a high impact on the field.

DESCRIPTION (provided by applicant): In the United States, mid-life adults are staying in the workforce longer and are engaging in higher levels of physical activity than previous generations, potentially extending their interactions with elements of the built and natural environments that influence their experience of daily stress. The relationship between various types of stress and alterations in cognitive functioning has been well documented, and there is growing evidence showing a relationship between daily stress in mid-life and cognitive decline. Even though mid-life adults constitute one of the largest portions of the current U.S. population, there is relatively little data about the experience of daily stress and health outcomes in this age group. Perceived stress in mid-life adults has also been closely linked with physical disability later in life. Mid-life adults of different socioeconomic status and race are subject to structural inequality in their living environments. Those living in urban environments with higher levels of poverty and violence are experience higher levels of environmental stress than those living in suburban or rural environments. It is well documented that Blacks are exposed to environmental stressors more than Whites. In this Stage 0 study of the NIH Stage Model for Behavioral Intervention Development, we propose to use our novel custom-built Stress Reports in Free-living Environments (STRIVE) ecological momentary assessment (EMA) mobile phone app to measure the experience of daily stress of mid-life adults in free-living conditions. Using our novel app and capturing data in real time will allow us to determine (1) where and when daily stress happens for mid-life adults, (2) whether mid-life adults’ daily stressors are linked to certain elements of the built and natural environment, and (3) whether EMA measurement of daily stress is comparable to the ‘gold standard’ Daily Inventory of Stressful Events (DISE) measurement tool that captures daily stress at the end of the day in mid- life adults (used in Midlife in the United State Survey (MIDUS)). These data will expand our understanding of mid-life adults’ experience of stress in free living conditions and pave the way for data-driven individual and community-based intervention designs to promote health and well-being in mid-life adults. Potential interventions that could stem from our data include technology based ‘just in time’ interventions that account for spatiotemporal location, mindfulness-based interventions that

incorporate environmental stress triggers, and community-based interventions designed to eliminate environmental stressors that are commonly experienced by mid-life adults (e.g., congested areas).

PUBLIC HEALTH RELEVANCE: The experience of daily stressors can accelerate cognitive decline and increase the risk of heart disease, depression, and other debilitating chronic illnesses – especially for those who are in underrepresented minority groups or live in low-income areas. In this project, we measure the daily stressors of mid-life (aged 50-64) adults using mobile phones, which allows us to better understand where and how stressors happen as people go about their daily lives. This knowledge can be used to develop community or psychological interventions to address environmental stressors and promote healthy aging.

CRITIQUE 1

Significance: 2
Investigator(s): 1
Innovation: 1
Approach: 2
Environment: 3

Overall Impact: This resubmission seeks to determine where and when daily stress occurs for mid-life adults in the built and natural environment, which environmental factors play a role in stress, and how certain groups (e.g., minorities, low income) are disparately impacted by the environments in which they live among 300 mid-life (age 50-64) adults from the greater Indianapolis area of Indiana. The long-term goal of proposed work is advancing the understanding of the environmental features that encourage or interfere with healthy aging to inform potential interventions aimed at removing environmental stressors and promoting activities in healthy environments. The significance of the topic and innovative combination of measurement tools are distinct strengths of the application. The research team is well-qualified, and appropriate analytical methodology has been proposed to achieve the aims of the study. However, reviewers noted weaknesses that lowered enthusiasm for the proposal, including a weak physical activity measure; lack of measurement for substance use; lack of generalizability; data collection at work; lack of data about validity and reliability of stress measures; potential safety issues related to taking photos; absence of documented community-based support; lack of income based sampling; and no clear plan for ensuring that most salient indicators of stress would lead to intervention. While investigators responded to a number of concerns, there are weaknesses that have not been addressed, so even with the significant and timely nature of the proposal, its potential to generate robust and responsive evidence that adds substantially to the field is not completely clear.

1. Significance:

Strengths

- In the United States, mid-life adults are staying in the workforce longer and are engaging in higher levels of physical activity than previous generations, potentially extending their interactions with elements of the built and natural environments that influence their experience of daily stress.
- The experience of daily stress can be detrimental to the health of mid-life adults, and research has shown that stress can accelerate cognitive decline and increase the risk of heart disease, depression, and other debilitating chronic illnesses.
- Stress has been particularly associated with places like home, work, and urban environments.

- Natural environments such as parks or the woods are associated with reduced perceived stress, and spending time in natural environments can create a stress ‘buffer’, reducing overall stress arousal even after leaving.
- Neighborhoods with high poverty rates and higher proportions of racial or ethnic minorities have less access to green space and higher exposure to stressful environmental elements like abandoned buildings and crime.
- Knowledge about where and when daily stress occurs can be used by community planners to address areas in the built and natural environment where stress happens most often, community organizations to provide support during high stress time periods within those locations, healthcare providers to make recommendations for healthy behaviors, and mid-life adults to make decisions about their movements in the built and natural environment to reduce daily stress.
- Little is known about the real life experience of daily stress, as past research has collected data primarily using end-of-day recall, which is subject to recall error and bias.
- Lack of naturalistic, real-world stress data from mid-life adults, which limits the ability to use stress research to inform health recommendations, treatments, or interventions.
- This project is expected to yield a more comprehensive understanding of (1) where and when daily stress happens for mid-life adults and (2) whether daily stressors are attributable to certain elements of the built and natural environment.
- Preliminary analysis of data collected in two pilot studies indicate that real time method of measuring daily stress results in greater capture of daily stressors.

Weaknesses

- Indiana sample is not representative of the adult US population.

2. Investigator(s):

Strengths

- PI has experience implementing ecological momentary assessment of stress using mobile and wearable technology in the context of tourism.
- PI has overseen two preliminary studies, one in a tourism context, and one that identically matches currently proposed methods.
- Study team has complementary expertise in physical activity and quality-of-life outcomes; sociotechnical systems and mechanisms to support health and wellbeing and reduce health disparity of marginalized and underserved populations; a range of methodologies, including lab-based experimental studies, daily diary studies, longitudinal observational studies, and controlled intervention trials to examine how stress in a variety of forms influences emotion, cognition, and cellular functioning; epidemiologic, statistical, and geospatial modeling techniques; and extensive experience in designing and conducting clinical and public health studies focusing on various adverse outcomes as a function of where individuals live, specifically aspects of neighborhood-based research using advanced epidemiological methods (e.g., bias correction, propensity scores), a geographic information system (GIS), and spatial statistics.
- Blossoming funding and publication records.

Weaknesses

- None noted by reviewer.

3. Innovation:

Strengths

- Innovative combination of measurement tools, including a smartphone based ecological momentary assessment app, ambulatory ECG monitor, and mixed methods analysis including spatiotemporal models and qualitative thematic analysis.
- Advances in mobile and wearable technology now allows for capturing in the moment accounts of stress, heart rate variability, and geospatial location simultaneously.
- Capturing these data in real time can help reduce recall bias and allow for a better understanding of where stress occurs in the built and natural environment. In the moment measurements of daily stress in free-living conditions also allows examination of intraday stress, which is unable to be captured by popular daily recall methods of measuring daily stress. Capturing in the moment stress data will also allow investigators to attribute stressors directly to elements of the environment in which they occur, rather than other variables such as participant activities.

Weaknesses

- None noted by reviewer.

4. Approach:

Strengths

- Multi-method, transdisciplinary approach to measuring the psychological (*in the moment* self-reports) and physiological (heart rate variability (HRV)) experience of stress in free-living conditions for mid-life adults.
- Use of a custom-built smartphone-based ecological momentary assessment (EMA) app and ambulatory electrocardiogram (ECG) recorder.
- EMA app passively collects global positioning system (GPS) coordinates of research participants several times per minute in addition to participant self-reports of daily stressors, including subjective severity.
- Ambulatory ECG monitor records heart rate 24-hours per day at 1ms resolution.
- Spatiotemporal analysis of reported stressful events and HRV data will allow investigators to determine where research participants are (and aren’t) when they experience stress in the built and natural environment.
- Participant reports of daily stressors via EMA app will include a short description of the stressful experience and a picture of the stressful environment for context, allowing for qualitative thematic analysis of text- and image-based stressor data and providing rich detail in understanding of stressors and the role the environment played in stressful experiences.
- 300 mid-life adults will be recruited to participate in the study for one week on a rolling basis.
- Purposive sampling to ensure a minimum of half of research participants are members of minority groups.

Weaknesses

- No conceptual model presented that is matched with the selection of variables to guide assessment and evaluation in the study.

5. Environment:

Strengths

- Appear to have necessary resources at respective institutions to carry out proposed work
- Leverages lab that designs, prototypes, and deploys novel personal health informatics devices, interfaces, and platforms to support people with physical, developmental, and mental conditions.
- Investigative team has developed relationships with local public health organizations focused on minority populations (e.g., Minority Health Coalition of Marion County) who have committed to aid in recruitment of those in minority groups.

Weaknesses

- Investigators report that several local public health organizations have committed to share recruitment via social media, but there are NO community-based letters of support included.

Protections for Human Subjects:

Acceptable Risks and/or Adequate Protections

Data and Safety Monitoring Plan (Applicable for Clinical Trials Only):

Acceptable

Inclusion Plans:

- Sex/Gender: Distribution justified scientifically
- Race/Ethnicity: Distribution justified scientifically
- Inclusion/Exclusion Based on Age: Distribution justified scientifically

Vertebrate Animals:

Not Applicable (No Vertebrate Animals)

Biohazards:

Not Applicable (No Biohazards)

Resubmission:

- Investigators have substituted a chest worn ambulatory electrocardiogram (ECG) monitor for a Polar Unite smartwatch in the proposal. The Polar watch is a much less invasive and more practical tool for measuring ambulatory heart rate (via photoplethysmography (PPG)) and activity level (via accelerometer). The HR and activity level measurements collected via the Polar smartwatch will be incorporated into the spatiotemporal model as covariates.
- Added Aim 3, which compares number of stressors, stressor type, and subjective severity of stressors between STRIVE and the DISE. This analysis will show how stressor data collected by these two tools are similar and different.

- Adjusted sampling strategy to include a 2 (underrepresented minorities, non-underrepresented minorities) x 3 (low-, middle-, and high-income) stratification.
- Secured a letter of support from the Indiana Clinical and Translational Sciences Institute (CTSI) committing their recruitment concierge services team will aid in the recruitment of study participants via the All IN for Health volunteer registry and community partnerships.
- Added a table (Table 2) that summarizes all study variables that will be collected in the pre-test survey, real time, and end of day recall.
- Added the details of IRB approved protocol with instructions for reporting stressors in potentially dangerous situations and instructions for taking pictures of stressful environments.
- Now include pretest measures of childhood stress (ACES) as well as other common stressors (e.g., financial strain) to account for these stressors.
- Expanded the data collection period to three-weeks (2 weeks for each measurement tool with 1 week of overlap) to obtain a more robust sample of stressors.
- Pre-test survey now includes some measures of interpersonal stress (e.g., social provisions scale).

Resource Sharing Plans:

Acceptable

Budget and Period of Support:

Recommend as Requested

CRITIQUE 2

Significance: 3
Investigator(s): 2
Innovation: 2
Approach: 3
Environment: 2

Overall Impact: This resubmission R21 application is from a strong interdisciplinary group of investigators that seeks to measure stress in real time and space, and to tie these measures to the places in the built and natural environment in which they occur. The methods to be used are innovative. The team has provided data to support the methods. The topic is important because daily stress contributes to physical and mental health outcomes. There is potential to develop targeted interventions and/or treatments because the data provides information in real time and space. The investigative team addressed many (if not all) of the minor to moderate weaknesses noted in the first submission, including a new measure of objective physical activity, survey data on substance use and abuse, and inclusion of a NIA recommended tool for measuring daily stressors. Overall, the proposed research will provide important data on novel methods for measuring stress in real time and space, and such data can lead to actionable ways of handling stress encountered in daily life in the future.

1. Significance:

Strengths

- Spatiotemporal data collection that can lead to actionable recommendations, treatments, or interventions.
- Plans to enhance recruitment of populations particularly exposed to stress.

Weaknesses

- The previous lack of perceived and/or actual stress measures in early life has largely been addressed, although the measures included are imperfect and reflect primarily adverse childhood events and current financial strain. (minor weakness)

2. Investigator(s):

Strengths

- PI is an ESI with experience specific to the application's focus (stress in the built and natural environment) as documented through a few publications and 3 grants.
- Co-Is provide additional expertise.

Weaknesses

- None noted by reviewer.

3. Innovation:

Strengths

- Subjective and objective measures of stress where and when they occur in daily life.
- Use of tools to capture and analyze data within a spatiotemporal framework.

Weaknesses

- The DISE tool is an imperfect "gold (alloy) standard" of daily stress, but it is replicable. (minor weakness).

4. Approach:

Strengths

- Comprehensive list of confounding variables that will be measured and included in statistical models.
- Robust spatiotemporal modeling.

Weaknesses

- The need for participants to use their smartphones to report daily stressors in the moment is itself a stressor – this could lead to "fatigue" in that participants may not wish to report all stressors they encounter to reduce burden on themselves. (minor weakness)
- Still not clear what land cover use data ISDP provides and what variables will be created from such data – will this be used for a measure of green space akin to NDVI? – noted on page 51 that this data "will permit us to determine characteristics of the built/natural environment such as tree canopy cover, parks, residential green spaces and designated land uses ……. for each timestamp location." (minor weakness)

5. Environment:

Strengths

- More than adequate for the proposed research.

Weaknesses

- None noted by reviewer.

Protections for Human Subjects:

Unacceptable Risks and/or Adequate Protections

- Participants should be made aware that knowledge of their geolocation is a potential risk (i.e., confidentiality) and should be provided the opportunity to turn off instrumentation when they are in a place they perceive as sensitive (this is similar to items describing guidelines about when it is and is not ok to take a picture of stressful environments).

Inclusion Plans:

- Sex/Gender: Distribution justified scientifically
- Race/Ethnicity: Distribution justified scientifically
- Inclusion/Exclusion Based on Age: Distribution justified scientifically
- Justified mid-life adults only based on specific aims and hypotheses of a specific FOA (PA18-850: Prevention Research in Mid-Life Adults)

Vertebrate Animals:

Not Applicable (No Vertebrate Animals)

Biohazards:

Not Applicable (No Biohazards)

Resubmission:

- The investigators provide a detailed explanation of changes to the A1 proposal. Importantly, they have decided to do away with an HRV measure and instead include a tool to measure HR and activity levels continuously. Other major changes include inclusion of a reliable tool for measuring stress (an "alloy" standard) and improved sampling and recruitment strategies.

Resource Sharing Plans:

Acceptable

Budget and Period of Support:

Recommend as Requested

CRITIQUE 3

Significance: 2
Investigator(s): 4
Innovation: 2
Approach: 3
Environment: 1

Overall Impact: This proposal addresses a very significant issue in the measurement of daily stressors with a focus on spatial-temporal correlates. Overall impact is moderately high because the research is likely to provide evidence of the validity and feasibility of a measurement tool for improved understanding of contextual mechanisms linked to stress and stress-related health outcomes in mid-life adults. The project is innovative, because it provides evidence for an improved measurement technique that be applied to interventions in this population. The investigative team has appropriate and complementary skills and the environment is excellent. The approach has some very strong aspects such as stratified sampling and excellent plan for recruiting. Most of the weaknesses were minor. However, one weakness created some concern. The weakness was lack of evidence for the team's plan and support for triangulating physiological stress with psychological stress. The problem with this is that they do not provide hypotheses or analyses related to this and the investigator identified with this expertise is not included in project (e.g., no biosketch). Additionally, the unprecedented plan for long-term monitoring of participants and management of large quantities of data not credibly addressed. In conclusion, despite the weakness in the approach, the potential overall impact of this project remains high because it will advance understanding of the mechanisms underlying the relationship between neighborhood context and stress-related health issues and test new methods that will be useful in both epidemiological and translational research areas.

1. Significance:

Strengths

- Use of STRIVE to identify daily stressors in the moment and place they occur connected with environmental context including crime, greenspace, and landuse is an exciting opportunity to better characterize exposure and identify mechanisms for stress-related health outcomes.
- Preliminary data support the feasibility and value of using the STRIVE app; proposed project will add information about the value added compared to existing end of day survey.
- Research credibly links the anticipated study findings regarding evidence of daily stressors that are relevant for this population to the development of evidence-based individual and community interventions to address health and well-being in mid-life adults.

Weaknesses

- Proposal provides no convincing evidence that majority of daily stress related to environmental exposure.
- Although proposal identifies importance of work for mid-life adults, aims do not directly address relevant contextual work-related information (e.g., stress occurring inside workplace).

2. Investigator(s):

Strengths

- Investigators have complementary expertise needed to conduct the proposed project (measurement of stress, EMA, psychological stress, activity/heart rate, GIS.

Weaknesses

- Schootman provides expertise to integrate heart rate and activity levels into spatiotemporal analyses and interpretation for Aim 1 but no biosketch is included.
- Plan for coordination of transdisciplinary team not clear.

3. Innovation:

Strengths

- Use of EMA to more effectively link stress to environmental exposures over time is innovative.

Weaknesses

- Integration of passive data collection (of heart rate and activity) when examining the impact of stress on well-being is potentially innovative but this aspect is not well developed (not clear that project has aims, methods to fully contribute).

4. Approach:

Strengths

- Stratified sampling approach ensures adequate representation by SES and race/ethnicity.
- Excellent plan for recruiting participants; ALL IN volunteer registry is available to investigators with adequate number of non-white volunteers to help ensure successful recruitment to identified strata; strong letter of support from Indiana Clinical and Translational Sciences Institute (CTSI).
- Well designed protocols.
- Analyses appropriate to address aims.

Weaknesses

- Minor concern regarding participant burden given the long study period and intensive requirements has not been adequately addressed and may lead to unanticipated drop-out.
- Moderate concern regarding feasibility/process of data management for unprecedented amounts of data collected in this study not adequately addressed.
- Moderate concern regarding analyses/hypotheses related to triangulation of physiological stress with psychological stress not provided thus limits the likely significance of the findings.
- Minor concern regarding Workplace identified but not directly addressed in terms of reporting/capturing information.

5. Environment:

Strengths

- Resources, facilities, and equipment appropriate for the needs of the proposed project including management and analysis of data generated from EMA.

Weaknesses

- None noted by reviewer.

Protections for Human Subjects:

Acceptable Risks and/or Adequate Protections

- Acceptable

Inclusion Plans:

- Sex/Gender: Distribution justified scientifically
- Race/Ethnicity: Distribution justified scientifically
- Inclusion/Exclusion Based on Age: Distribution justified scientifically
- Acceptable

Vertebrate Animals:

Not Applicable (No Vertebrate Animals)

Biohazards:

Not Applicable (No Biohazards)

Resubmission:

- Responsive to prior comments. Specifically, changed stratified sampling plan to ensure adequate variability in participants, added third aim to compare EMA tool (STRIVE) to recommended daily measure (DISE), substituted less invasive data collection for heart rate and activity, and provided additional information regarding feasibility of recruitment.

Budget and Period of Support:

Budget Modifications Recommended (in amount/time)

Recommended budget modifications or possible overlap identified:

- BSC does not identify data management as a responsibility in this project. Research assistants only in Y1 (relatively limited amount of time given the large population and need for recruitment/data collection; no research assistants in Y2 to provide support for data management. Schootman not identified in budget justification.

THE FOLLOWING SECTIONS WERE PREPARED BY THE SCIENTIFIC REVIEW OFFICER TO SUMMARIZE THE OUTCOME OF DISCUSSIONS OF THE REVIEW COMMITTEE, OR REVIEWERS' WRITTEN CRITIQUES, ON THE FOLLOWING ISSUES:

PROTECTION OF HUMAN SUBJECTS: UNACCEPTABLE. The protection of human subjects from research risks is unacceptable. See Reviewer 2 comments. This needs to be addressed.

INCLUSION OF WOMEN PLAN: ACCEPTABLE

INCLUSION OF MINORITIES PLAN: ACCEPTABLE

INCLUSION ACROSS THE LIFESPAN: ACCEPTABLE

COMMITTEE BUDGET RECOMMENDATIONS: Recommended budget modifications. See reviewer 3 comments.

Footnotes for 1 R21 AG077333-01A1; PI Name: Jordan, Evan

+ Derived from the range of percentile values calculated for the study section that reviewed this application.

NIH has modified its policy regarding the receipt of resubmissions (amended applications).See Guide Notice NOT-OD-18-197 at https://grants.nih.gov/grants/guide/notice-files/NOT-OD-18-197.html. The impact/priority score is calculated after discussion of an application by averaging the overall scores (1-9) given by all voting reviewers on the committee and multiplying by 10. The criterion scores are submitted prior to the meeting by the individual reviewers assigned to an application, and are not discussed specifically at the review meeting or calculated into the overall impact score. Some applications also receive a percentile ranking. For details on the review process, see http://grants.nih.gov/grants/peer_review_process.htm#scoring.

MEETING ROSTER

Community Influences on Health Behavior Study Section
Healthcare Delivery and Methodologies Integrated Review Group
CENTER FOR SCIENTIFIC REVIEW
CIHB
06/06/2022 - 06/07/2022

Notice of NIH Policy to All Applicants: Meeting rosters are provided for information purposes only. Applicant investigators and institutional officials must not communicate directly with study section members about an application before or after the review. Failure to observe this policy will create a serious breach of integrity in the peer review process, and may lead to actions outlined in NOT-OD-22-044 at https://grants.nih.gov/grants/guide/notice-files/NOT-OD-22-044.html, including removal of the application from immediate review.

CHAIRPERSON(S)

RAMASWAMY, MEGHA, PHD
PROFESSOR
DEPARTMENT OF POPULATION HEALTH
SCHOOL OF MEDICINE
UNIVERSITY OF KANSAS
KANSAS CITY, KS 66160

MEMBERS

BEAMER, PALOMA I, PHD
PROFESSOR
DEPARTMENT OF COMMUNITY, ENVIRONMENT AND POLICY
MEL AND ENID ZUCKERMAN COLLEGE OF PUBLIC HEALTH
UNIVERSITY OF ARIZONA
TUCSON, AZ 85724

BONAR, ERIN E, PHD
ASSOCIATE PROFESSOR
DEPARTMENT OF PSYCHIATRY
UNIVERSITY OF MICHIGAN
ANN ARBOR, MI 48109

CHEN, ANGELA CHIA-CHEN, PHD *
ASSOCIATE PROFESSOR
EDSON COLLEGE OF NURSING AND HEALTH INNOVATION
ARIZONA STATE UNIVERSITY
PHOENIX, AZ 85004

CORLISS, HEATHER L, PHD
PROFESSOR
DIVISION OF HEALTH PROMOTION
AND BEHAVIORAL SCIENCE
SCHOOL OF PUBLIC HEALTH
SAN DIEGO STATE UNIVERSITY
SAN DIEGO, CA 92123

DRABBLE, LAURIE A, PHD
PROFESSOR
COLLEGE OF HEALTH AND HUMAN SCIENCES
SAN JOSE STATE UNIVERSITY
SAN JOSE, CA 95192

DUNCAN, GLEN E, PHD
PROFESSOR AND CHAIR
DEPARTMENT OF NUTRITION AND EXERCISE PHYSIOLOGY
ELSON S. FLOYD COLLEGE OF MEDICINE
WASHINGTON STATE UNIVERSITY
SPOKANE, WA 99202

GOHLKE, JULIA M, PHD
ASSOCIATE PROFESSOR
DEPARTMENT OF POPULATION HEALTH SCIENCES
VIRGINIA POLYTECHNIC INSTITUTE
AND STATE UNIVERSITY
BLACKSBURG, VA 24061

GOODKIND, JESSICA R, PHD
ASSOCIATE PROFESSOR
DEPARTMENT OF SOCIOLOGY
UNIVERSITY OF NEW MEXICO
ALBUQUERQUE, NM 87131

HARVEY, IDETHIA SHEVON, DRPH, MPH *
ASSOCIATE PROFESSOR
SCHOOL OF HEALTH PROFESSIONS
UNIVERSITY OF MISSOURI
COLUMBIA, MO 65211

KARASZ, ALISON, PHD *
ASSOCIATE PROFESSOR
DEPARTMENT OF FAMILY AND SOCIAL MEDICINE
MONTEFIORE MEDICAL CENTER
ALBERT EINSTEIN COLLEGE OF MEDICINE
BRONX, NY 10467

KRISTJANSSON, ALFGEIR, PHD *
ASSOCIATE PROFESSOR
DEPARTMENT OF SOCIAL AND BEHAVIORAL SCIENCES
SCHOOL OF PUBLIC HEALTH
WEST VIRGINIA UNIVERSITY
MORGANTOWN, WV 26506

LIU, BIAN, PHD *
ASSOCIATE PROFESSOR
DEPARTMENT OF POPULATION HEALTH SCIENCE AND POLICY
ICAHN SCHOOL OF MEDICINE AT MOUNT SINAI
NEW YORK, NY 10029

MARSHALL, BRANDON D, PHD
ASSOCIATE PROFESSOR
DEPARTMENT OF EPIDEMIOLOGY
SCHOOL OF PUBLIC HEALTH
BROWN UNIVERSITY
PROVIDENCE, RI 02912

MASON, MICHAEL J, PHD
BETSEY R BUSH ENDOWED PROFESSOR
CENTER FOR BEHAVIORAL HEALTH RESEARCH
COLLEGE OF SOCIAL WORK
UNIVERSITY OF TENNESSEE
KNOXVILLE, TN 37996

MASSEY, PHILIP M, PHD
ASSOCIATE PROFESSOR
DEPARTMENT OF HEALTH, HUMAN PERFORMANCE AND RECREATION
UNIVERSITY OF ARKANSAS
FAYETTEVILLE, AR 72701

MICHAEL, YVONNE LOUISE, SCD *
PROFESSOR
DEPARTMENT OF EPIDEMIOLOGY AND BIOSTATISTICS
SCHOOL OF PUBLIC HEALTH
DREXEL UNIVERSITY
PHILADELPHIA, PA 19102

MOJTABAI, RAMIN, MD, MPH, PHD
PROFESSOR
DEPARTMENT OF MENTAL HEALTH
BLOOMBERG SCHOOL OF PUBLIC HEALTH AND
DEPARTMENT OF PSYCHIATRY AND BEHAVIORAL SCIENCES
JOHNS HOPKINS UNIVERSITY
BALTIMORE, MD 21205

NIKULINA, VALENTINA, PHD *
ASSISTANT PROFESSOR
DEPARTMENT OF PSYCHOLOGY
QUEENS COLLEGE
QUEENS, NY 11367

PAHL, KERSTIN, PHD
RESEARCH ASSOCIATE PROFESSOR
DEPARTMENT OF PSYCHIATRY
SCHOOL OF MEDICINE
NEW YORK UNIVERSITY
NEW YORK, NY 10016

PELTIER, RICHARD E, MPH, PHD *
ASSOCIATE PROFESSOR
DEPARTMENT OF ENVIRONMENTAL HEALTH SCIENCE
SCHOOL OF PUBLIC HEALTH AND HEALTH SCIENCES
UNIVERSITY OF MASSACHUSETTS AMHERST
AMHERST, MA 01003

PERKINS, MOLLY M, PHD *
ASSOCIATE PROFESSOR
DIVISION OF GERIATRICS AND GERONTOLOGY
DEPARTMENT OF MEDICINE
SCHOOL OF MEDICINE
EMORY UNIVERSITY
ATLANTA, GA 30322

QUINTILIANI, LISA M, PHD
ASSOCIATE PROFESSOR
SCHOOL OF MEDICINE
BOSTON UNIVERSITY
BOSTON, MA 02118

RONIS, SARAH DIANE, MD, MPH, PHD *
ASSISTANT PROFESSOR
DEPARTMENT OF PEDIATRICS AND ADOLESCENT MEDICINE
SCHOOL OF MEDICINE
CASE WESTERN RESERVE UNIVERSITY
CLEVELAND, OH 44106

ROY, BRITA, MD, MPH *
ASSISTANT PROFESSOR
DEPARTMENT OF INTERNAL MEDICINE
SCHOOL OF MEDICINE
YALE UNIVERSITY
NEW HAVEN, CT 06510

SCARINCI, ISABEL C, MPH, PHD
PROFESSOR
DEPARTMENT OF OBSTETRICS AND GYNECOLOGY
UNIVERSITY OF ALABAMA AT BIRMINGHAM
BIRMINGHAM, AL 35294

SHADEL, WILLIAM G, PHD *
SENIOR BEHAVIORAL SCIENTIST
RAND CORPORATION
PITTSBURGH, PA 15213

THRASHER, JAMES, PHD
PROFESSOR
DEPARTMENT OF HEALTH PROMOTION, EDUCATION AND BEHAVIOR
ARNOLD SCHOOL OF PUBLIC HEALTH
UNIVERSITY OF SOUTH CAROLINA
COLUMBIA, SC 29208

TUITT, NICOLE R, DRPH, MPH *
ASSISTANT PROFESSOR
CENTERS FOR AMERICAN INDIAN AND ALASKA
NATIVE HEALTH
DEPARTMENT OF COMMUNITY AND BEHAVIORAL HEALTH
UNIVERSITY OF COLORADO ANSCHUTZ MEDICAL CAMPUS
DENVER, CO 80045

WARD, TONY JOHN, PHD
PROFESSOR AND CHAIR
SCHOOL OF PUBLIC
AND COMMUNITY HEALTH SCIENCES
UNIVERSITY OF MONTANA
MISSOULA, MT 59812

WILLIAMS, EDITH MARIE, PHD
ASSOCIATE PROFESSOR
DIVISION OF RHEUMATOLOGY
DEPARTMENT OF PUBLIC HEALTH SCIENCES
MEDICAL UNIVERSITY OF SOUTH CAROLINA
CHARLESTON, SC 29425

ZALLER, NICKOLAS D, PHD *
PROFESSOR
DIVISION OF HEALTH BEHAVIOR AND HEALTH EDUCATION
COLLEGE OF PUBLIC HEALTH
COLLEGE OF PROVOST
UNIVERSITY OF ARKANSAS FOR MEDICAL SCIENCES
LITTLE ROCK, AR 72205

MAIL REVIEWER(S)

EMBRY, LEANNE, PHD
PROFESSOR
DEPARTMENT OF PEDIATRICS
DIVISION OF PEDIATRIC HEMATOLOGY-ONCOLOGY
JOE R. AND TERESA LOZANO LONG SCHOOL OF MEDICINE
UNIVERSITY OF TEXAS HEALTH SCIENCE CENTER
SAN ANTONIO, TX 78229

KOHL, PATRICIA LYNN, PHD
ASSOCIATE PROFESSOR
GEORGE WARREN BROWN SCHOOL OF SOCIAL WORK
WASHINGTON UNIVERSITY IN ST. LOUIS
ST. LOUIS, MO 63130

SHI, YUYAN, PHD
ASSOCIATE PROFESSOR
DEPARTMENT OF FAMILY MEDICINE AND PUBLIC HEALTH
UNIVERSITY OF CALIFORNIA, SAN DIEGO
LA JOLLA, CA 92093

SCIENTIFIC REVIEW OFFICER

PELTOLA, PIA KRISTIINA, PHD
SCIENTIFIC REVIEW OFFICER
CENTER FOR SCIENTIFIC REVIEW
BETHESDA, MD 20892

EXTRAMURAL SUPPORT ASSISTANT

SOTO, CASSANDRA LYNNE
LEAD EXTRAMURAL SUPPORT ASSISTANT
CENTER FOR SCIENTIFIC REVIEW
NATIONAL INSTITUTES OF HEALTH
BETHESDA, MD 20892

* Temporary Member. For grant applications, temporary members may participate in the entire meeting or may review only selected applications as needed.

Consultants are required to absent themselves from the room during the review of any application if their presence would constitute or appear to constitute a conflict of interest.
